# From in vitro research to real life studies: an extensive narrative review of the effects of balneotherapy on human immune response

**DOI:** 10.1007/s11332-021-00778-z

**Published:** 2021-05-20

**Authors:** M. C. Maccarone, G. Magro, U. Solimene, A. Scanu, S. Masiero

**Affiliations:** 1 Physical Medicine and Rehabilitation School, University of Padova, Via Giustiniani 3, 35128 Padua, Italy; 2 World Federation of Hydrotherapy and Climatotherapy, Milan, Italy; 3 Reumathology Unit, Department of Medicine – DIMED, University of Padova, Padua, Italy; 4 Rehabilitation Unit, Department of Neuroscience, University of Padova, Padua, Italy

**Keywords:** Spa therapy, Health resort therapy, Mud therapy, Peloidotherapy, Immune system

## Abstract

**Purpose:**

The biologic mechanisms by which balneotherapy (BT) alleviates symptoms of different diseases are still poorly understood. Recently, preclinical models and clinical trials have been developed to study the effects of BT on the immune system. This review summarizes the currently available evidence regarding the effects of spa therapy on the immune response, to confirm the role of BT in the enhancement of immune system and open interesting research fields.

**Methods:**

PubMed and Google Scholar were searched from 1997 up to June 2020, with search criteria including terms related to BT and immune system. We selected only in vitro research, randomized controlled trials (RCTs) or clinical trials.

**Results:**

In vitro studies on human and animal samples have demonstrated that thermal waters exert anti-inflammatory and immunomodulatory effects. In particular, H_2_S donors seem to counteract the inflammatory processes in psoriatic lesions, arthritic fibroblast-like synoviocytes and chondrocytes, and regulate important factors implicated in osteoarthritis pathogenesis and progression. RCTs and clinical trials revealed, after BT, a reduction in circulating levels of pro-inflammatory molecules, such as TNF-α, IL-1β, and C-reactive protein, and an increase in anti-inflammatory molecules such as the IGF-1 growth factor especially in musculoskeletal diseases.

**Conclusion:**

Further preclinical studies and RCTs could help to exploit BT in real life for preventive and therapeutic treatments.

## Introduction

In the era of COVID-19 pandemic, the importance of the immune system has become central, becoming a topic of great interest in several fields of medicine. It is interesting to know whether health resort therapy, one of the most commonly used complementary therapies, especially as mud and baths applications, can affect the immune system. In recent years, there has been an increased interest in the use of preclinical models (in vitro studies on human or animal samples) to investigate the biological effects of balneotherapy (BT) on inflammation and immunity. Recently, also clinical trials and Randomized Controlled Trials (RCTs) have been developed to study the effects of BT on the human immune system.

BT includes immersion in thermal waters, balneological interventions with natural gases, mud applications and other traditional remedies [1]. The treatment in the spa setting is frequently supplemented by other interventions including health education and health promotion strategies [2]. Many mineral-rich waters have been confirmed to have effects on the immune system and recent findings suggest that BT and mud therapy may act on the immune response even if the mechanisms are still not completely understood [3, 4]. Our aim is to summarize the current available information about the effects of thermal mineral waters or of their organic and inorganic components on the immune response not only to confirm the important role of BT in the enhancement of human immune system but also to open interesting further fields of research.

## Materials and methods

A scoping review was conducted with the aim of searching for the evidence of BT effects on the immune responses and the immune system from 1997. First, the research question was defined by the first author in collaboration with the other authors. Then, a bibliographic research was carried on Medline (PubMed) and Google Scholar. As keywords, we used spa therapy, health resort medicine, balneotherapy, mud therapy, immune response, immunity, immune system combined employing the Boolean operators. Studies were eligible if they were in vitro research on human or animal samples, randomized controlled trials (RCTs) or clinical trials; BT or mineral-rich water or mud applications had to be the intervention under study and had to be compared with another intervention or with no intervention. A comprehensive process of identifying and selecting appropriate studies was conducted.

Studies selected for the review needed to have the abstract available and published in English. Articles with the abstract written in languages other than English were excluded.

All original research articles published from 1997 up to June 2020 were analyzed.

Case reports, letters to the editor, and studies published before 1997 were excluded. Also repetitions and studies not related to the topic were excluded.

At the end of the selection process, 40 studies were elegible for the scoping review.

After the identification of relevant studies, the data were extracted and charted. Papers that did not meet the inclusion criteria were excluded. The first author and his assistants independently screened the papers and downloaded the full-text versions. The authors' names, the year of publication, the journal, the pathology investigated, the study design or the experimental model, the population, the age of the participants, the kind of water/mud utilized, the presence of a comparison group, the outcomes evaluated, and the significance of the results were extracted and gathered in comprehensive tables.

## Results

### In vitro studies on human samples evaluating the beneficial effects of BT on immune response in skin diseases

Most of the in vitro studies on human samples evaluating the immunological effects of thermal waters in dermatology focused on psoriasis. Psoriasis is an immune-mediated, inflammatory disease, in which intralesional T lymphocytes induce keratinocytes to proliferate and perpetuate the disease process. The interleukins IL-17 and IL-22 produced by Th1/Th17 lymphocytes stimulate IL-8 secretion by keratinocytes, and this represents a key event in the psoriasis pathogenesis [5]. Sulfur is able to penetrate the skin, and sulfur-rich waters used in BT may be effective in the treatment of psoriasis. Evaluating the effects of thermal water rich in sodium hydrosulfide (NaHS) on human psoriatic keratinocytes, Gobbi et al. [6] and Mirandola et al. [7] demonstrated a reduction in psoriasis lesions through a decrease in IL-8, IL-17 and IL-22.

Since skin psoriatic manifestations are thought to be angiogenesis-dependent, effects on the expression and release of vascular endothelial growth factor (VEGF)-A were studied by Chiarini et al. [8] using Comano’s thermal water, rich in sodium, calcium and bicarbonate. They demonstrated, evaluating cultured human lesional keratinocytes exposed to thermal water, that BT interferes with VEGF-A expression and secretion. Also, a reduction in IL-6 levels and in the expression of cytokeratin-16, a marker associated with keratinocyte psoriatic phenotype, was demonstrated [9]. Finally, a reduction in the intracellular and secretion levels of IL-8 and tumor necrosis factor (TNF)-α, pro-inflammatory cytokines overexpressed in psoriatic lesions, was shown using the same water [10].

Karagülle et al. in 2018 [11], evaluating human psoriasis and rosacea keratinocytes treated with Bursa and Bolu thermal mineral waters showed a reduction of inflammation and neo-angiogenic phenomena (reduced IL-1α, TNF-α, and VEGF gene expression).

Lee et al. [12] evaluated the effects of thermal water on human keratinocytes related to skin immune reactions. The treatment with thermal water decreased the expression of pro-inflammatory cytokines, such as IL-6, IL-8, IL-1α, TNF-α, and granulocyte–macrophage colony-stimulating factor (GM-CSF). In addition, the differentiation process of subsets of T helper cells, such as Th1, Th2 and Th17 cells (related to autoimmunity or to inflammatory skin diseases), was reduced. Thermal water induced also Foxp3^+^T_reg_ cell differentiation in vitro, implying that the immunomodulatory effect also includes T_reg_ cell-induced immune suppressive effects.

All the treatment details and the results of these studies are reported in Table 1.

**Table 1:** In vitro studies on human samples evaluating the beneficial effects of BT on immune response in skin diseases

Authors	Journal	Pathology	Experimental model	Treatment	Mineral water or inorganic or organic components	Biochemicalparameters	Results
Gobbi et al. 2009 [6]	Lab Investig	Psoriasis	Normal skin-derived immortalized human keratinocytes	30-min preincubation with MAPK/ERK inhibitors (10–30 μM) + NaHS (400 mM) dissolved in the culture medium, for 6, 12, 18, 24 h	NaHS	Cell proliferation and adhesion; MAPK/ERK signaling Phosphorylation	↓keratinocyte cell growth and adhesion ↓MAPK/ERK phosphorylation ↓inflammation
Mirandola et al. 2011 [7]	Lab Investig	Psoriasis	Normal skin-derived immortalized human keratinocytes	30-min preincubation with MAPK/ERK inhibitors (10–30 μM) + NaHS (400 mM) dissolved in the culture medium, for 6, 12, 18, 24 h	NaHS	IL-8; MAPK/ERK signaling phosphorylation	↓ basal and IL-17/IL-22-induced IL-8 expression and secretion ↓ MAPK/ERK phosphorylation ↓ inflammation
Chiarini et al. 2006 [8]	Int J Mol Med	Psoriasis	Human primary epidermal keratinocytes	25%, 50%, or 100% of Comano water dissolved in the culture medium, for 11 days	Comano spa’s water (Trentino, Italy), rich in sodium, calcium and bicarbonate	VEGF-A	↓ VEGF-A ↓ VEGF-A-mediated effects
Chiarini et al. 2006 [9]	Int J Mol Med	Psoriasis	Human primary epidermal keratinocytes	25%, 50%, or 100% of Comano water dissolved in the culture medium, from 3 to 15 days	Comano spa’s water (Trentino, Italy), rich in sodium, calcium and bicarbonate	IL-6, CK-16, VEGF-A	↓ IL-6, VEGF, and CK-16 release and expression ↓ inflammation, and neo-angiogenic phenomena
Dal Pra et al. 2007 [10]	Int J Mol Med	Psoriasis	Human primary epidermal keratinocytes	25%, 50%, or 100% of Comano water dissolved in the culture medium, from 11 to 13 days	Comano spa’s water (Trentino, Italy), rich in sodium, calcium and bicarbonate	IL-8, TNF-α	↓ IL-8 and TNF-α ↓ inflammation
Karagülle et al. 2018 [11]	Int J Biometeorol	Psoriasis and rosacea	Human keratinocyte cell lines	10% of Bursa and Bolu thermal mineral waters dissolved in the culture medium, for 3 days		IL-1α, TNF-α, and VEGF	↓ IL-1α, TNF-α, and VEGF gene expression ↓ inflammation, and neo-angiogenic phenomena
Lee et al. 2012 [12]	Ann Dermatol	Skin disease	Human keratinocyte cell lines	50% of Yong-gung oncheon thermal spring water dissolved in the culture medium, for 1, 4, 10, and 24 h + LPS (10 μL/mL)	HaCaT Thermal spring water (Yong-gung oncheon, Ganghwa-gun, Korea), rich in sulfur, magnesium, calcium and selenium	IL-6, IL-8; CD4 + T cell differentiation	↓ IL-6 and IL-8 gene and protein expression ↓ differentiation of CD4 + T cells in Th1, Th2 and Th17 ↓ immune skin reactions

*CK* cytokeratin, *ERK* extracellular signal-regulated kinase, *IL* interleukin, *LPS* lipopolysaccharide, *MAPK* mitogen-activated protein kinase, *NaHS* sodium hydrosulfide, *Th* T helper, *TNF* tumor necrosis factor, *VEGF* vascular endothelial growth factor, *↓* decrease

### In vitro studies on human samples evaluating the beneficial effects of BT on immune response in musculoskeletal diseases

Immunological effects of BT in musculoskeletal diseases have been most widely studied in rheumatic disorders. Fox et al. [13] studied the ability of human primary chondrocytes and mesenchymal progenitor cells to synthesize hydrogen sulfide (H_2_S) in response to pro-inflammatory stimulation and their response to an exogenous slow-releasing H_2_S source, the morpholin-4-ium 4 methoxyphenyl (morpholino) phosphinodithioate (GYY4137). Endogenous H_2_S produced by the cells and the treatment with GYY4137 significantly reduced cell death and oxidant-induced mitochondrial dysfunction, caused by inflammatory cytokines.

Li et al. [14] assessed the effect of GYY4137 on lipopolysaccharide (LPS)-dependent release of inflammatory mediators from human arthritis synoviocytes and articular chondrocytes. GYY4137 demonstrated an anti-inflammatory effect decreasing the production of nitrite, prostaglandin E2 (PGE2), TNF-α, and IL-6 from both cell types, reducing the levels and the catalytic activity of inducible nitric oxide synthase (iNOS) and cyclooxygenase (COX)-2, and nuclear factor kappa B (NF-κB) activation induced by LPS. Similar results were obtained by Burguera et al. [15] Ha et al. [16] in studies on human osteoarthritis (OA) chondrocytes stimulated with IL-1β, a pro-inflammatory cytokine, to reproduce the “OA-like effect”. Burguera et al.’s study was also the first to demonstrate the anti-catabolic activity of these compounds through the downregulation of metalloproteinase (MMP)-13. Also, Fioravanti et al. [17] studying the potential beneficial effect of Vetriolo thermal water (Trentino Alto Adige, Italy), a highly mineralized water, strongly acidic sulfate, rich in calcium, magnesium, and iron, in human OA chondrocytes, showed after the treatment a significant reduction in NO levels and in the expression of iNOS.

Sieghart et al. [18] showed that the NaHS treatment in OA fibroblast-like synoviocytes reduced spontaneous and IL-1β-induced secretion of IL-6, IL-8, the expression of MMP-2 and MMP-14, and the phosphorylation of several mitogen-activated protein kinases (MAPKs). Later, a similar research was conducted on OA cartilage extracts co-cultured with IL-1β and NaHS or GYY4137 [19]. At the end of the treatment, there was a reduction of the catabolic processes (decrease in glycosaminoglycan destruction and MMP-3 and MMP-13 production) and a stimulation of the cell anabolism (increased synthesis of collagen type II alpha 1 chain and aggrecans).

Kloesch et al. [20, 21] evaluated the effects of NaHS on fibroblast-like synoviocytes derived from rheumatoid arthritis (RA) and OA patients. IL-1β-induced expression of IL-6 was transiently and partially down-regulated with low concentrations of NaHS. Long-term exposure to H_2_S and high concentration of NaHS provided stimulatory effects, leading to the reinforced activation of p38 MAPK and extracellular signal-regulated kinases (ERK)1/2 accompanied by upregulation of IL-6 expression. In 2012, Kloesch et al. [22] demonstrate a reduction of IL-6 and IL-8 expression and the activation of p38/MAPK/ERK and NF-kB signaling in a human chondrocyte cell line treated with NaHS.

On bone-derived cells, only a limited number of in vitro studies were performed. On human osteoclasts, after an incubation period with NaHS, a significant decrease in osteoclast differentiation and intracellular reactive oxygen species (ROS) levels, and an increased transcription of antioxidant genes were observed [23].

All the treatement details and the results of these studies are reported in Table 2.

**Table 2:** In vitro studies on human samples evaluating the beneficial effects of BT on immune response in musculoskeletal diseases

Authors		Journal		Pathology		Experimental model			Treatment		Mineral water or inorganic or organic components			Biochemicalparameters	Results
Fox et al. 2012 [13]		J Cell Mol Med		RA		Human primary articular chondrocytes			IL-1β, IL-6 and TNF-α (5 ng/mL) for 6, 12 and 18 h + GYY4137 (50–500 mol/L) dissolved in the culture medium, for 12 h		GYY4137			Cell death; Mitochondrial membrane potential	↓ cell death and oxidant-induced mitochondrial dysfunction ↓ inflammation
Li et al. 2013 [14]		J Cell Mol Med		RA		Human primary arthritis synoviocytes and chondrocytes			(0.1–0.5 mM) dissolved in the culture medium, for 18 h + LPS (10 μg/mL)		GYY4137				↓ IL-6, TNF-α, PGE2 and NO production ↓ COX-2 and iNOS catalytic activity ↓ NF-kB activation ↓ inflammatory processes
Burguera et al. 2014 [15]		Osteoarthr Cartil		OA		Human primary OA chondrocytes			NaHS and GYY4137 (0.05–1 mM) dissolved in the culture medium, for 24 or 48 h + IL-1β (5 ng/mL)		NaHS and GYY4137			IL-6, PGE2, PTGES, COX-2; NO, NOS2; MMP-13; NF-kB signaling activation	↓ IL-6, PGE2, and NO release and protein level ↓ IL-6, PTGES, COX-2, and NOS2 gene expression ↓ NF-κB nuclear translocation ↓ inflammatory and degrading processes
Ha et al. 2015 [16]		Int J MolMed		OA		Human primary OA chondrocytes			NaHS (0.06–1.5 mM) dissolved in the culture medium, for 24 h + IL-1β (10 ng/mL)		NaHS			COX-2, iNOS, MMP-13; ERK/IκBα/NF-κB signaling activation	↓ COX-2, iNOS, MMP-13 release and gene expression ↓ERK/IκBα/NF-κB activation ↓ degrading processes
Sieghart et al. 2015 [18]		J Cell Mol Med				Human primary OA fibroblast-like synoviocytes			NaHS (0.06–1 mmol/L) dissolved in the culture medium, for 1 h + IL-1β (10 ng/mL)		NaHS			IL-6, IL-8; MMP-2, MMP-14; MAPK and Akt1/2/PI3K protein phosphorylation	↓ IL-6 and IL-8 secretion, MMP-2 and MMP-14 gene expression ↓ MAPK phosphorylation ↑ Akt1/2 phosphorylation ↓ inflammatory and degrading processes
Vela-Anero et al. 2017 [19]		Nitric Oxide		OA		Human OA cartilage disks			NaHS or GYY4137 (200 or 1000 μM) dissolved in the culture medium, for 21 days + IL-1β (5 ng/mL)		NaHS and GYY4137			MMP-3, MMP-13; COL2A1, glycosaminoglycans, aggrecans	↓ MMP-3 and MMP-13 production ↑ COL2A1, glycosaminoglycans and aggrecans synthesis ↓ degrading processes
Fioravanti et al. 2013 [17]		J Biol Regul Homeost Agents		OA		Human primary OA chondrocytes			25%, 50%, or 100% of Vetriolo thermal water dissolved in the culture medium, for 48 h + IL-1β (5 ng/mL)		Vetriolo thermal water (Trentino Alto Adige Italy), strongly acidic sulfate, rich in calcium, magnesium and iron			NO, iNOS; Cell viability and apoptosis; Morphological assessment	25%, 50% of Vetriolo water ↑ survival recovery rate ↓ NO levels, iNOS expressions, and apoptosis ↑ morphological characteristics ↓ degrading processes
Kloesch et al. 2010 [20]	Cell Biol Int			RA	fibroblast-like synoviocytes			NaHS (200 mM)		NaHS			IL-6; activation/deactivation of MAPKs; p38 and p44/42 MAPK (ERK1/2)		↓ IL-1β-induced expression of IL-6 with low concentrations of NaHS H_2_S deactivates p44/42 MAPK (ERK1/2) ↑ activation of p38 MAPK, ERK1/2 and IL-6 with long-term exposure to H_2_S
Kloesch et al. 2012 [21]	Immunol Lett		RA and OA		RA and OA human fibroblast- like synoviocytes		NaHS (1.0 mM) dissolved in the culture medium, for 1, 3, 6, 12 h			NaHS		IL-6, IL-8, COX-2; MMP-2, MMP-3, MMP-14; p38/MAPK/ERK protein expression			↑ IL-6, IL-8, COX-2 and p38/MAPK/ERK expression
Kloesch et al. 2012 [22]	Rheumatol Int		RA		Human chondrocyte cell line		NaHS (0.125 and 1.0 mM) dissolved in the culture medium, for 15, 30, 45 and 60 min + MAPK inhibitors (1 and 5 μM) + IL-1β(5 ng/mL) for 1 h			NaHS		IL-6, IL-8; P38/MAPK/ERK and NF-kB signaling activation/- deactivation			↓ IL-6 and IL-8 ↓ p38/MAPK/ERK ↓ NF-kB signalling ↓ inflammatory processes
Gambari et al. 2014 [23]		Pharmacol Res		Osteoporosis		Human differentiated osteoclasts			NaHS (50–300 μM) dissolved in the culture medium, for 72 h to 6 days		NaHS			Osteoclasts differentiation; ROS production, NRF2, KEAP1, NQO1, and PRDX1	↓ osteoclast differentiation ↓ intracellular ROS levels ↑ NRF2 protein expression and nuclear translocation ↑ antioxidant gene

*Akt* protein-chinasi B*, COL2A1* collagen type II alpha 1 chain, *COX* cyclooxygenase, *ERK* extracellular signal-regulated kinase, *GYY4137* morpholin-4-ium 4 methoxyphenyl (morpholino) phosphinodithioate, *IκBα* inhibitor of nuclear factor kappa B, *IL* interleukin, *iNOS* inducible nitric oxide synthase, *KEAP1* kelch like ECH associated protein 1, *LPS* lipopolysaccharide, *MAPK* mitogen-activated protein kinase, *MMP* matrix metalloproteinase, *NaHS* sodium hydrosulfide, *NF-kB* nuclear factor kappa B, *NO* nitric oxide, *NOS2* nitric oxide synthase 2, *NQO1* NAD(P)H quinone dehydrogenase 1, *NRF2* nuclear factor-erythroid factor 2-related factor 2, *OA* osteoarthritis, *PGE2* prostaglandin E2, *PI3K* phosphoinositide 3-kinase, *PRDX1* peroxiredoxin 1, *PTGES* prostaglandin E synthase, *RA* rheumatoid arthritis, *ROS* reactive oxygen species, *TNF* tumor necrosis factor, *↓* decrease, *↑* increase

### In vitro studies on animal samples evaluating the beneficial effects of BT on immune response in musculoskeletal diseases

Xu et al. [24] showed the proliferative, antioxidant, and anti-inflammatory effects of a treatment with NaHS in hydrogen peroxide-stimulated murine osteoblast-like cell line. The antioxidant effects were confirmed by Lv et al. [25], in an analogous experimental study, examining the effect of GYY4137 added at the culture medium for 4 h in the presence of hydrogen peroxide (H_2_O_2_).

On rat primary osteoblasts cultured with NaHS, after an incubation with high glucose concentration, NaHS significantly prevented osteoblast injury induced by glucose, blocking the glucose-induced osteoblast mineralization inhibition [26].

All the treatment details and the results of these studies are reported in Table 3.

**Table 3:** In vitro studies on animal samples evaluating the beneficial effects of BT on immune response in musculoskeletal diseases

Authors	Journal	Pathology	Experimental model	Treatment	Mineral water or inorganic or organic components	Biochemicalparameters	Results
Xu et al. 2011 [24]	Free Radic Biol Med	Osteoporosis	Murine osteoblast-like cell line (MC3T3-E1)	NaHS (100 μM) dissolved in the culture medium, for 4 h + (H_2_O_2_) (400 μM)	NaHS	Viability, proliferation and apoptosis; NO, ALP, SOD, NADPH oxidase p38/ERK1/2/MAPKs activation	↑ viability ↑ cell proliferation ↑ ALP and SOD activities ↓ apoptosis ↓ NO release ↓ NADPH oxidase activity ↓ p38/ ERK1/2/ MAPKs activation Proliferative and antioxidant effects against osteoporosis damage
Lv et al. 2017 [25]	Am J Transl Res	Osteoporosis	Murine osteoblast-like cell line (MC3T3-E1)	GYY4137 (100 μM) dissolved in the culture medium, for 4 h + (H_2_O_2_) (400 μM)	GYY4137	Viability, proliferation, Runx2, and apoptosis; NO, ALP, and SOD, ERK1/2 activation	↑ viability ↑ cell proliferation ↑ ALP and SOD activities ↑ Runx2 gene expression ↓ apoptosis ↓ NO release ↓ ERK1/2 activation Proliferative and antioxidant effects
Liu et al. 2017 [26]	Biochimie	Osteoporosis	Rat primary osteoblasts	NaHS (400 μmol/L) dissolved in the culture medium, for 12 h	NaHS	Osteoblast proliferation and mineralization; Apoptosis; KATP protein expression	↓ cell proliferation ↑ apoptotic cells ↑ osteoblast mineralization ↑ KATP protein expression ↓ osteoporosis damage

*ALP* alkaline phosphatase, *ERK* extracellular signal-regulated kinase, *GYY4137* morpholin-4-ium 4 methoxyphenyl (morpholino) phosphinodithioate, *H*_*2*_*O*_*2*_ hydrogen peroxide, *KATP* ATP-sensitive potassium, *MAPK* mitogen-activated protein kinase, *NADPH* nicotinamide adenine dinucleotide phosphate, *NaHS* sodium hydrosulfide, *NO* nitric oxide, *Runx2* runt-related transcription factor 2, *SOD* superoxide dismutase, *↓* decrease, *↑* increase

### In vitro studies on human samples evaluating the beneficial effects of BT on immune response in inflammatory diseases

The protective effects of H_2_S and of its exogenous sources on cellular immune response were first investigated by Rinaldi et al. [27] in a study on purified human neutrophils, eosinophils, and lymphocytes of human donors treated with NaHS at different concentrations for 24 h. After the treatment, an increased short-term survival of neutrophils was found, while no changes in lymphocytes and eosinophils were observed. A similar experimental protocol was performed by Mirandola et al. [28] in peripheral blood lymphocytes purified from healthy subjects. After the incubation with NaHS, a decreased proliferation of CD8 + T and natural killer (NK) cells and reduced IL-2 production were observed. Sulen et al. [29] stimulated human peripheral blood mononuclear cells with NaHS, showing after the treatment the phosphorylation of p38, protein kinase B (Akt), and cAMP-response element-binding protein (CREB).

The proliferative activity of H_2_S donors was also demonstrated in a study carried out on peripheral blood lymphocytes isolated from patients with systemic lupus erythematosus. H_2_S donors inhibited the abnormal activation and proliferation of lupus lymphocytes through the Akt/glycogen synthase kinase 3 beta (GSK3β) pathway [30].

In activated human neutrophils treated with sulfurous water of Acqui Terme (Piemonte, Italy), a significant reduction of ROS and reactive nitrogen species (RNS) was observed after the treatment [31]. A similar experiment conducted treating neutrophils with different concentrations of the above mentioned sulfurous water or NaHS demonstrated the inhibition of elastase release, revealing a possible contribution in controlling the inflammatory processes of airway diseases [32].

In human primary monocytes treated with NaHS, the production of TNF-α, IL-1β, IL-6, IL-12, and C–C motif chemokine ligand 5 (CCL5) induced by LPS was blocked and ROS formation and antioxidant enzymes activity were reduced. Sirmione thermal water (Lombardia, Italy), rich in sodium chloride, bromide, and iodide, did not show the same results, and enhanced the release of IL-10, probably due to the low concentration of sulfur compounds [33].

Given the important role of Th17/Treg cell ratio in the onset and evolution of immune-mediated pathologies, in 2018, Vitale [34] investigated the effects of exogenous H_2_S on human CD4 T cell polarization to Th17 and/or Treg phenotype. NaHS treatment increased both Foxp3 mRNA levels in CD4 + T cells under Treg-polarizing conditions and retinoic acid-related orphan receptor gamma t (RORγT) mRNA levels in CD4 + T cells under Th17 polarizing conditions, suggesting a role of sulfur in both polarization pathways.

All the treatment details and the results of these studies are reported in Table 4.

**Table 4:** In vitro studies on human samples evaluating the beneficial effects of BT on immune response in inflammatory diseases

Authors	Journal	Treatment	Experimental model	Mineral water or inorganic or organic components	Pathology	Biochemicalparameters	Results
Rinaldi et al. 2006 [27]	Lab Investig	NaHS (from 0.23 to 3.66 mM) dissolved in the culture medium, for 24 h + p38/MAPK inhibitors (30–60 μM)	Human purified neutrophils, eosinophils or lymphocytes	NaHS	Inflammatory processes of respiratory tract	Cell viability and apoptosis; p38/MAPK signaling activation/deactivation	↑ Short-term survival of neutrophils ↓ caspase-3 cleavage and p38/MAPK phosphorylation in neutrophils Accelerate the resolution of inflammatory processes
Mirandola et al. 2007 [28]	J Cell Physiol	NaHS (from 0.20 to 4.0 mM) dissolved in the culture medium, for 24 h + caspase inhibitors (30 μM)	Human purified peripheral blood lymphocytes	NaHS	Inflammatory processes	Cell viability and apoptosis; IL-2	↓ proliferation of lymphocyte subsets ↓ D8 + T and NK cells ↓ IL-2 production Accelerate the resolution of inflammatory processes
Sulen et al. 2016 [29]	Pharmacol Res	NaHS (10, 100 or 1000 μM) dissolved in the culture medium, for 10 min	Human peripheral blood mononuclear cells (PBMCs)	NaHS	Inflammatory processes	p38/MAPK, NF-κB p65, Akt and CREB phosphorylation	↑ p38/MAPK, Akt, and CREB phosphorylation ↓ inflammatory processes
Han et al. 2013 [30]	Cell Physiol Biochem	NaHS (0.25, 0.5, 1, 2, 4 and 8 mM) and GYY4137 (200, 400, 800, 1600 μM) dissolved in the culture medium, for 0.5, 1, 2, 4, 6, 12, 24, 36, 48 h	Human purified peripheral blood lymphocytes	NaHS and GYY4137	Inflammatory processes of systemic lupus erythematosus	Cell viability, cell cycle distribution; Akt (ser473), GSK3β (ser9), p27Kip1 and p21CIP1	↑ cell proliferation and S phase distribution of cell cycle ↓ Akt (ser473), GSK (ser9) ↑ p27Kip1 and p21CIP1 expression and phosphorylation ↓ inflammatory processes
Braga et al. 2008 [31]	Respiration	Sulfurous thermal water (different concentrations) dissolved in the culture medium, for 15 min + N-formyl-methionyl-leucyl-phenylalanine/phorbol-12-myristate-13-acetate	Human purified neutrophils	Sulfurous thermal water (Acqui Terme, Piemonte, Italy), which contains different HS groups concentrations	Inflammatory processes	ROS and RNS	↓ ROS and RNS release at 0.94 to 15.5 μg/mL of HS ↓ inflammatoryprocesses
Braga et al. 2010 [32]	TherAdvRespirDis	Sulfurous water or NaHS (from 4.5 to 18 mg/mL) dissolved in the culture medium, for 15 min	Human purified neutrophils	Sulfurous thermal water (AcquiTerme, Piemonte, Italy) and NaHS	Inflammatory processes of upper and lower airway diseases	Elastase release; elastolyticactivity	Inhibited elastase release ↓ inflammatory processes
Prandelli et al. 2013 [23]	Int J ImmunopatholPharmacol	Sirmione thermal water or NaHS (2.5 mM) dissolved in the culture medium, for 24 h + LPS (100 ng/mL)	Human primary monocytes	Sirmione thermal water (Lombardia, Italy), rich in sodium chloride, bromine and iodine	Chronic inflammatory and age-related illness	TNF-α, IL-1β, IL-6, IL-12; CXCL8, CCL5; ROS, antioxidant enzymes	↓ TNF-α, IL-1β, IL-6, IL-12, CXCL8, CCL5 production ↓ ROS formation and antioxidant enzymes Sirmione water ↑ IL-10 release ↓ chronic inflammatory and age-related illness manifestations
Vitale 2018 [34]	Bol Soc Esp Hidrol Méd	Exogenous H2S on human resting CD4 T cell polarization to Th17 and/or Treg phenotype	Differentiated ex-vivo human resting CD4 + (Th0) T cells to Th17 or Treg lineages	NaHS	Immune-mediated pathologies	CD4 T cell polarization to Th17 and/or Treg phenotype	↑ Foxp3 mRNA levels in CD4 + T cells cultured under Treg-polarizing conditions ↑ RORγT mRNA levels in CD4 + T cells under Th17 polarizing conditions

*AKT* protein-chinasi B, *CCL5* C–C motif chemokine ligand 5, *CREB* cAMP-response element-binding protein, *CXCL8* C-X-C motif chemokine ligand 8, *GSK3β* glycogen synthase kinase 3 beta, *HS* sulfhydryl, *IL* interleukin, *LPS* lipopolysaccharide, *MAPK* mitogen-activated protein kinase, *NaHS* sodium hydrosulfide, *NF-κB* nuclear factor kappa B, *NK* natural killer, *RNS* reactive nitrogen species, *ROS* reactive oxygen species, *Th* T helper, *TNF* tumor necrosis factor, *Treg* regulatory T, *↓* decrease, *↑* increase

### In vitro studies on animal samples evaluating the beneficial effects of BT on immune response in inflammatory diseases

H2S can have a role also as an endogenous and exogenous immunomodulatory molecule in T cells signal in inflammatory bowel diseases. H_2_S donors employed to treat primary mouse T lymphocytes (CD3 +) and CD4 + T cells enhanced T cell activation, IL-2 expression, and CD25 levels. Besides, activation increased the capacity of T cells to synthesize endogenous amounts of H_2_S through the increased expression of cystathionine γ-lyase and cystathionine β-synthase [35].

These results are reported in Table 5.

**Table 5:** In vitro studies on animal samples evaluating the beneficial effects of BT on immune response in inflammatory diseases

Authors	Journal	Treatment	Experimental model	Mineral water or inorganic or organic components	Pathology	Biochemicalparameters	Results
Miller et al. 2012 [35]	J Biol Chem	H_2_S (50–500 nM) dissolved in the culture medium, for 4, 10, and 24 h	Primary mouse T lymphocytes (CD3 +), OT-II CD4 + T cells	H_2_S	Inflammatory processes of bowel diseases	CD69, CD25; IL-2; cystathionine γ-lyase, cystathionine β-synthase	↑ T cell activation ↑ CD69 and IL-2 expression ↑ CD25 levels ↑ cystathionine γ-lyase and cystathionine β-synthase expression ↓ inflammatory processes

*H*_*2*_*S* hydrogen sulfide,* IL* interleukin, *↓* decrease, *↑* increase

### Clinical trials and RCTs evaluating the beneficial effects of BT on immune response

Most of the clinical studies and RCTs evaluating the immunological effects of thermal waters focused on musculoskeletal diseases. Bellometti et al. in 1997 [36] conducted a study enrolling a group of 22 OA patients. The patients underwent 12 mud pack treatments and after the treatment an increase in insulin growth factor 1 (IGF-1) and a decrease of TNF-α in serum were observed. After mud pack therapy also, a decrease in serum levels of PGE2 and leukotriene (LTB4), was observed [37]. In patients with primary symptomatic bilateral knee OA, randomly assigned to receive a cycle of mud-bath therapy or to continue their standard therapy alone, in the group of mud-bath therapy, a significant increase of C-terminal cross-linked telopeptide type II collagen (CTX-II), perhaps due to an increase of cartilage turnover induced by thermal stress, was observed [38]. Ortega et al. [39] in 2017 evaluated the effects of a 10-day cycle of mud therapy in a group of patients with primary knee OA. After the cycle of mud therapy, serum concentrations of IL-1β, TNF-α, IL-8, IL-6, TGF-β, and extracellular heat-shock protein 72 (eHsp72) were markedly decreased and systemic levels of cortisol significantly increased.

Galvéz et al. [40] showed in patients with knee OA who underwent a cycle of BT with mud applications a reduction in the percentage of CD4 + T regulatory cells and an enancement in CD8 + T regulatory cells, wich play a key role in regulating immune reactions, contolling inflammation and maintaining immune homeostasis. In addition, an increased neutrophil functional capacity was observed.

Tarner et al. [41] analyzed the effect of mild whole-body hyperthermia in ankylosing spondylitis (AS). Serum samples were taken to measure TNF-α, IL-1β and IL-6. Hyperthermia caused a significant reduction of all cytokines by 40–50%. A significant increase in transforming growth factor (TGF)-β1 was found in AS patients treated with active exercises, hyperthermia and exposure to low doses of radon in a former mine (total and active) [42].

In fibromyalgia, female patients treated with BT five days per week for 3 weeks, mean PGE2 levels were higher compared to healthy control group and decreased after the treatment period. Also, IL-1 and LTB4 significantly decreased after the treatment. [43]

Eysteinsdoóttir et al. [44] investigated the effects of bathing in geothermal seawater in addition to the narrow-band ultraviolet B (NB-UVB) therapy in patients suffering from psoriasis. Compared with healthy controls, psoriasis patients with active disease had significantly higher proportion of peripheral cutaneous lymphocyte-associated antigen (CLA) + T cells expressing C–C motif chemokine receptor 10 (CCR10) and CD103 and T cells with both Th1/Tc1 and Th17/Tc17 phenotypes. A reduction in circulating CLA + peripheral blood T cells and a decreased Th1/Th17 and Tc1/Tc17 inflammatory response were shown after BT and NB-UVB therapy.

The radioactive and thermal effects of radon hot spring were biochemically compared under a sauna room or hot spring conditions with a similar chemical component [45]. The radon and thermal therapy enhanced the antioxidation functions, such as the activities of superoxide dismutase and catalase, which inhibit lipid peroxidation and total cholesterol produced in the body, increased the percentage of CD4-positive cells (marker of helper T cells) and decreased the percentage of CD8-positive cells (marker of killer T cells and suppressor T cells). Furthermore, the therapy increased the levels of alpha atrial natriuretic polypeptide, beta endorphin, adrenocorticotropic hormone, insulin and glucose-6-phosphate dehydrogenase, and decreased the vasopression level. The results were larger in the radon group than in the thermal group, suggesting that radon therapy contributes more to the prevention of life-style-related diseases related to peroxidation reactions and immune suppression than thermal therapy.

All the treatment details and results of these studies are reported in Table 6.

**Table 6:** RCTs and clinical trials evaluating the beneficial effects of BT on immune response

First Author	Journal	N	Pathology	Age	Intervention	Treatment	Comparison	Type of water/inorganic or organic component	Systemic inflammatory Biomarkers	Stress biomarkers	Results
Bellometti et al. 1997 [36]	Clinica Chimica Acta	22	OA	63.6 year	Mud pack treatments	12 applications of Mature thermal mud to the whole body for 20 min at 40 °C, followed by a bath for 10–12 min at 37–38 °C	Yes (22 control group only hot bath)	Mud of Abano-Montegrotto Terme	Serum IGF-1 and TNF-α		Differences between mean values found before and after the mud pack statistically significant
Bellometti et al. 1998 [37]	J Investig. Med	31	Healthy		Mud pack Therapy				PGE2 and LTB4		↓ PGE2 and LTB4 in serum
Pascarelli et al. 2016 [38]	IMAJ	103	OA	68.49 ± 9.0	Mud bath therapy	2 weeks daily local mud-packs and baths	Control group (*n* = 50) regular care routine (exercise, symptomatic drugs, intra-articular hyaluronic acid)	Water of the Chianciano Spa Resort (Siena, Italy)	COMP, CTX-II, MPO and hsCRP in serum		↔ COMP, MPO and hsCRP in serum of either group ↑ in CTX-II serum levels in the mud-bath group after the treatment
Ortega et al. 2017 [39]	Int J Biometeorol	21	OA knee	62–77	Whole body Peloidotherapy	1 session/die × 10	No	SiO_2_, CaO, Al_2_O_3_ and Fe_2_O_3_	IL-1β, TNF-α, IL-8, IL-6, TGF-β	SerumCortisol, eHsp72	↓ serum concentrations of IL-1β, TNF-α, IL-8, IL-6 and TGF-β ↑ systemic levels of cortisol ↓ circulating levels of eHsp72
Galvéz et al. 2018 [40]	International journal of hyperthermia	36	Knee OA	70	Whole-body mud therapy at 40–42 °C	10-day cycle	No	SiO_2_, CaO, Al_2_O_3_ and Fe_2_O_3_	IL-8, TGFb, CD4, CD 25, FOXP3, CD28, CD8, Neutrophils		↓ circulating concentrations of IL-8 and TGF-β ↓ CD4 CD25 FOXP3 Treg cells ↑ CD8þ CD28– Treg cell ↑ neutrophils and phagocytic activity
Tarner et al. 2009 [41]	ClinicalRheumatology	12	AS	Meanage 33.4 years	Whole-body hyperthermia full bath	9 cycles (initial water temperature of 36 °C that was increased gradually by 1 °C every 5 min to 40 °C. This temperature was maintained until the body temperature reached 38.5 °C)	Yes (12 healthy control)	nd	Serum levels of the cytokines TNF-α, IL-1B e IL-6		↓ TNF-α, IL-1β, and IL-6 ↔ 1 h after baseline, ↓6 h after baseline ↓At 24 h after the treatment
Shehata et al. 2006 [42]	The Middle European Journal of medicine	83	AS	30–73	Spa exercises therapy	3–4 weeks spa-exercise therapy—in addition, outdoor exercises, physiotherapy, hydro-therapy and massage	Yes (control group with 10 patients with LBP and same treatment, second control group with 10 patients AS and no treatment)	Radon concentration up to 4.5 nCi/l; temperature 38–41 °C; relative humidity 70–98%	TGF-β1		↑ total and active TGF-β1
Ardic F. 2007 [43]	Rheumatol Int	12	FibromyalgiaSyndrome	43	BT	Group 1 (*n* = 12) received bathing for 20 min a day, for 5 days per week for 3 week	Yes (9 group no treatment + 10 control group healthy women)	HCO_3_, SO_4_	ESR, CRP, RF, PGE2, LTB4 and IL-1α in serum (before and at the end of general period of therapy)		↓ serum PGE2 level ↓ IL-1 and LTB4
Eysteinsdóttir et al. 2013 [44]	Scand JImmunol	12	Psoriasis	36.7	Bathing in geothermal seawater	Bathing in geothermal seawater twice daily for at least 1 h combined with NB-UVB therapy 5 days per week for 2 weeks	Yes (Of the 12 patients enrolled, six received treatment and 6 were treated with NB-UVB therapy)		Chemokines, inflammatory cytokines, T cells and TLRs in the blood and skin samples were evaluated on enrollment and at 1, 3 and 8 weeks		↓ Th17 (IL-23R + CD4 + T cells) ↓ IL-23R expressed by these cells and their IL-17/IL-22 cytokine secretion ↓ Tc17 T cells (producing IL-17 and IL-22) after both of the treatments ↓ Th1 and Tc1 phenotype (IFN-c and TNF-α production)
Yamaoka et al. 2004 [45]	J Radiat. Res	15	Healthy	20–40	A hot bathroom with a high concentration of radon	On days 1, 3, 5, 8, and 10, the inhalation of vapor from each hot spring under a condition of high humidity (about 90%)	Yes (3 groups: radon, thermal, and control.)	The temperature was 36 °C, the radon concentration was 2,080 Bq/m^3^	C4 positive cells and CD8-positive cells		On days 5 and 10, ↑ CD4-positive cells ↓ CD8-positive cells in the radon group

*Al*_*2*_*O*_*3*_ aluminum oxide, *CaO* calcium oxide, *AS* ankylosing spondylitis, *BT* balneotherapy, *COMP* cartilage oligomeric matrix protein, *CRP* C-reactive protein, *CTX-II* C-terminal cross-linked telopeptide type II collagen, *eHsp72* extracellular heat-shock protein 72, *ESR* erythrocyte sedimentation rate, *Fe*_*2*_*O*_*3*_ ferric oxide, *FOXP3* forkhead box P3, *HCO*_*3*_ bicarbonate, *hsCRP* high-sensitivity C-reactive protein, *IFN-c* interferon-c, *IGF-1* insulin-like growth factor 1, *IL* interleukin, *LTB4* leukotriene B4, *MPO* myeloperoxidase, *NB-UVB* narrow-band ultraviolet B, *OA* osteoarthritis, *PGE2* Prostaglandin E2, *RF* rheumatoid factor, *SiO*_*2*_ silicon dioxide, *SO*_*4*_ sulfate, *TGF-β* transforming growth factor beta, *Th* T helper, *TLR* toll-like receptor, *TNF* tumor necrosis factor, *↓* decrease, *↑* increase, *↔* no change

## Discussion

In in vitro human samples, H_2_S donors (NaHS and GYY4137) were demonstrated to exert anti-inflammatory and anti-angiogenic effects, confirming the beneficial properties of mineral water sulfur components on psoriatic lesions. H_2_S sources seem to counteract the inflammatory processes both in arthritic fibroblast-like synoviocytes and chondrocytes, and in OA chondrocytes. All these findings provide new information about the anti-inflammatory, antioxidant, and anti-catabolic properties of H_2_S. In particular, H_2_S seems to act as a chondroprotective agent by regulating important factors implicated in OA pathogenesis and progression, and counteracting IL-1β pro-inflammatory signals that lead to cartilage destruction. The capacity of H_2_S donors to limit the oxidative stress damage was demonstrated also in cell lines of osteoblasts. Moreover, the sulfide compounds appear to regulate inflammation and immune response in human psoriatic keratinocytes and in purified human peripheral blood neutrophils, eosinophils and lymphocytes.

Even in the cohorts of patients suffering from OA, BT has demonstrated to have anti-inflammatory efficacy, modulating the cytokinic response and modifying the percentage of regulatory T cells in circulation. After BT and mud therapy, a reduction in serum levels of pro-inflammatory molecules, such as TNF-α, IL-1β, PGE2, LTB4 and C-reactive protein, and an increase in anti-inflammatory molecules such as the IGF-1 growth factor have been shown. Furthermore, a decrease in the concentration of MMP, involved in cartilage degradation, has been reported after mud therapy in OA patients, maybe as a consequence of the reduction in pro-inflammatory mediators that promote MMP secretion. BT contributes also to a modification in cellular immune response: after mud therapy, OA patients presented increased neutrophils’ levels and functional capacity [46].

Also in patients with fibromyalgia or AS, BT can influence the inflammatory mediators. In particular, in AS patients, whole-body hyperthermia and speleotherapy resulted in changes of the pro-inflammatory cytokine network.

Finally, BT should have also an anti-inflammatory role on healthy subjects.

Due to the heterogeneity of composition characteristics of the natural mineral water or mud used and to the different sample size and performed protocols considered in the clinical trials, it is not possible to define with certainty the BT effects on the immune system. However, the results showed some beneficial effects that can to some degree positively modify the inflammatory response.

## Conclusion

In conclusion, studies on in vitro samples could open the way to the scientific progress to develop further clinical studies and RCTs evaluating the effects of BT on the immune system, to exploit BT in real life for preventive, curative and rehabilitation treatments [47].
